# Emphasising Learning in Health Promotion Targeting Individuals With Intellectual Disabilities

**DOI:** 10.1111/jir.70091

**Published:** 2026-03-06

**Authors:** Elin Fägerstam, Kristin Alfredsson Ågren, Maria Kvarnström, Tove Törnqvist, Madeleine Abrandt Dahlgren, Ulrika Müssener

**Affiliations:** ^1^ Department of Health, Medicine, and Caring Sciences Linköping University Linköping Sweden

**Keywords:** decision‐making, developmental disabilities, health education, health promotion, intellectual disability

## Abstract

**Background:**

Individuals with intellectual disabilities (ID) face disproportionately poor health profiles, underscoring the need for targeted and tailored health promotion strategies. Increasing health‐related knowledge is essential for making lifestyle changes. However, difficulties associated with ID can affect the learning processes within health promotion, challenging professionals to apply various strategies to promote health. There is a lack of research exploring strategies perceived as meaningful and helpful in everyday health promotion informed by the lived experiences of individuals with ID and the insights of those who support them. The aim of this study is to explore strategies and organisational prerequisites for health promotion targeting individuals with ID by including the perspectives of individuals with ID, their significant others and professionals within healthcare, social services and educational systems.

**Methods:**

The study was conducted in the southeast of Sweden. Data collection included eight workshops involving 30 participants in total. Individuals with ID (*n* = 14) participated in two constellations: one group attended a series of three workshops, whereas the other group attended a single workshop. Support persons, including significant others and professionals (*n* = 16), were divided into four separate groups, attending one workshop each. All workshops were audio‐recorded and transcribed verbatim. The data were analysed using qualitative content analysis.

**Results:**

Three categories emerged during analysis: enabling informed decision‐making for health promotion, promoting health and well‐being through encouraging relations, and organisational factors influencing prerequisites for health promotion.

**Conclusions:**

Learning about health among individuals with ID appears to constitute a vital component of healthy decision‐making, and support persons play a central role in facilitating health‐related learning in everyday contexts. Prominent strategies for health promotion targeting individuals with ID include enabling informed decision‐making and fostering encouraging relationships. These strategies must be responsive to individual needs, grounded in everyday relationships and supported by organisational structures. Hindering organisational structures and limited health promotion knowledge among professionals may constrain these efforts. Strengthening professional capacity and organisational responsibility within health, social care and educational systems could enhance the conditions for equitable health promotion in this population.

## 1 Introduction

Noncommunicable diseases threaten global health World Health Organization (WHO) 2014; Abbafati et al. 2020). Individuals with intellectual disabilities (ID) are consistently identified as having poorer overall health profiles, earlier onset of chronic illness and a higher prevalence of comorbidities (van den Bemd et al. 2022; Wang et al. 2023; Tyrer et al. 2019). Most commonly these include cardiovascular diseases (van den Bemd et al. 2022; Wang et al. 2023; Flygare Wallén et al. 2018; Reppermund et al. 2020), circulatory and respiratory diseases (O'Leary, Cooper, and Hughes‐McCormack 2018; Hirvikoski et al. 2021; Tyrer et al. 2021; Truesdale et al. 2021), cancer (Reppermund et al. 2020), mental health conditions (Reppermund et al. 2020; Buckley et al. 2020) and metabolic conditions such as diabetes (van den Bemd et al. 2022; Flygare Wallén et al. 2018) and obesity (Flygare Wallén et al. 2018). These disparities are further exacerbated by lifestyle factors such as limited physical activity (Tyrer et al. 2019; Diaz 2020; Dairo et al. 2016) and unhealthy diets (Hoey et al. 2017). Together, adverse health profiles and lifestyle factors contribute to heightened vulnerability to preventable conditions and an increased risk of premature mortality among individuals with ID (Tyrer et al. 2019; Reppermund et al. 2020; O'Leary, Cooper, and Hughes‐McCormack 2018; Hirvikoski et al. 2021; Tyrer et al. 2021; Truesdale et al. 2021; Diaz 2020).

Despite the poor health profiles, individuals with ID face barriers to receiving equitable care (Bourne et al. 2021; Shea et al. 2022), including health promotion. Health promotion refers to proactive strategies and activities that enable individuals to improve their health, prevent illness and maintain well‐being, often by addressing social, behavioural, and environmental factors (McKinnon et al. 2021). Barriers to equitable health promotion are multifaceted and include attitudinal, informational, practical and logistical obstacles (Bourne et al. 2021; Shea et al. 2022; Doherty et al. 2020; Ali et al. 2013; Stone et al. 2023; Shady et al. 2022).

Health disparities are further compounded by structural limitations in how health promotion is organised and delivered for this population. Healthcare and social services professionals often lack sufficient knowledge on how to encounter individuals with ID in general (Bourne et al. 2021; Shea et al. 2022; Doherty et al. 2020; Ali et al. 2013; Stone et al. 2023; Shady et al. 2022) and in the context of health promotion in particular (Leser et al. 2018; Kuijken et al. 2016; Nijhof et al. 2024). In addition to knowledge gaps, the absence of health‐promoting policies, low prioritisation of health promotion and limited organisational resources and time contribute to inconsistent health promotion support for individuals with ID (Nijhof et al. 2024; O'Leary, Taggart, and Cousins 2018). Consequently, health‐promoting efforts frequently remain non‐inclusive, increasing the vulnerability of individuals with ID to poor health outcomes and exclusion from preventive efforts (Reppermund et al. 2020; Willems et al. 2018).

The poor health profiles among individuals with ID are further exacerbated by the intellectual and cognitive difficulties that affect practical, social and conceptual domains (American Psychiatric Association 2013), including challenges in communication, understanding health‐related information and making health‐related decisions (Shady et al. 2022; Vetter et al. 2022; Geukes et al. 2019). Individuals with ID often make use of their social networks for support in daily life and in making health‐promoting decisions and lifestyle changes (Vetter et al. 2022; Geukes et al. 2019). Thus, significant others, healthcare and social services professionals play a crucial role in health promotion (Leser et al. 2018; Kuijken et al. 2016; Nijhof et al. 2024; Hewitt et al. 2013).

An important aspect of health promotion, in order to meet the unique needs of individuals with ID, involves making deliberate efforts to increase knowledge concerning healthy lifestyle behaviours. This is of importance as behaviour change represents a key component of health promotion, and health behaviours are shaped by individual skills that can be developed through health education. Health education encompasses learning experiences that increase knowledge, influence motivation and strengthen health literacy (World Health Organization (WHO) 2021).

Intellectual and cognitive difficulties associated with ID can affect the learning process involved in health promotion, highlighting the need for tailored support to the abilities of individuals with ID (Bergström et al. 2014; Taggart and Cousins 2014). This challenges healthcare and social services professionals to apply a versatile set of strategies to facilitate health‐related learning (Bergström et al. 2014; Roll 2018; Martin et al. 2021) and healthy behaviour change (Maenhout et al. 2024). Strategies may include using simplified language, easy‐to‐read materials, communication aids and visualisation of key points (Bergström et al. 2014; Roll 2018; Martin et al. 2021); encouraging discussion, reflection and participation (Bergström et al. 2014; Martin et al. 2021; Maenhout et al. 2024); and providing structure, preparation and assistance and offering rewards (Bergström et al. 2014).

Although such strategies are commonly delivered as stand‐alone interventions, health promotion could be strengthened by embedding health behaviours into daily routines for individuals with ID (Kuijken et al. 2020). Particularly, there is a need for research that involves the perspectives of individuals with ID, as well as support persons such as their significant others and professionals within healthcare, social services and educational systems, to explore which strategies are perceived as meaningful and helpful in the context of everyday health promotion targeting individuals with ID. Addressing this gap in knowledge is crucial, as it has the potential to generate valuable insights into how such strategies can be meaningfully applied in health promotion efforts. Exploring and understanding these strategies could provide valuable lessons for enhancing communication and understanding between individuals with ID and their support persons. Such insights could lead to more personalised and effective health promotion that enables individuals with ID to make lasting lifestyle changes.

Furthermore, organisational resources and barriers are often underexplored in health promotion targeting individuals with ID (Kuijken et al. 2020). Understanding organisational prerequisites is essential, as the outcome of health promotion depends not only on organisational structures but also on the strategies used by support persons. This knowledge can inform the development of accessible health promotion efforts and support practices ultimately improving the quality, relevance and equity of health promotion provided to this population. Therefore, this study aims to explore strategies and organisational prerequisites for health promotion targeting individuals with ID by including the perspectives of individuals with ID, their significant others and professionals within healthcare, social services and educational systems.

## 2 Methods

This is a qualitative study (Patton 2015) using workshops for data collection. Transcribed data were analysed using qualitative content analysis (Lindgren et al. 2020; Graneheim et al. 2017). The Consolidated Criteria for Reporting Qualitative Research (COREQ) 32‐item checklist was applied (Tong et al. 2007). Ethical approval for the study was granted by the Swedish Ethical Review Authority (reference number: 2022‐06682‐01). All study procedures were conducted in accordance with the Declaration of Helsinki.

### 2.1 Study Context

This study is part of a larger research project, I DID IT (Intellectual Disability Integrating Digital Intervention Technology for health), which aims to promote healthy behaviours among individuals with ID. The study was conducted in the southeast of Sweden. Three laws govern care and support for individuals with ID in Sweden: the Health and Medical Services Act, the Social Services Act and the Act concerning Support and Service for Persons with Certain Functional Impairments (LSS) (Hälso‐ och sjukvårdslag (SFS 2017:30), n.d.; Socialtjänstlag (2025:400), n.d.; Lag om stöd och service till vissa funktionshindrade (SFS 1993:387), n.d.). Common LSS services include daily activity and supported housing (either group home with full‐time support or service homes with part‐time support) (Lag om stöd och service till vissa funktionshindrade (SFS 1993:387), n.d.; Socialstyrelsen 2024). These laws ensure equal rights and access to healthcare, social services and support. Responsibility for health promotion is shared by healthcare, social services and the educational system (Hälso‐ och sjukvårdslag (SFS 2017:30), n.d.; Socialtjänstlag (2025:400), n.d.; Lag om stöd och service till vissa funktionshindrade (SFS 1993:387), n.d.; Skollag (SFS 2010:800), n.d.).

### 2.2 Study Procedure and Recruitment

Recruitment and data collection were conducted by three researchers with a PhD degree: Female Researcher 1 (author Ulrika Mussener) with experience in qualitative methodology and health promotion, Female Researcher 2 (author Kristin Alfredsson Ågren) and Male Researcher 3 (author Stefan Johansson) with experience in research concerning participation and individuals with ID. No prior relationship was established between the researchers and participants before the commencement of the study.

A purposive sampling strategy (Patton 2015) was used to recruit participants, aiming for a diversity in age, gender and experience, as well as a broad range of expertise and perspectives from individuals with ID, healthcare, social services, educational systems, municipalities, disability interest organisations and significant others. Individuals with ID were eligible if they were at least 16 years old, able to communicate verbally in the workshops and had an interest in health promotion. Significant others and professionals were eligible if they were also over 16 years of age and had experience working with or supporting individuals with ID.

Participants were recruited through established networks within healthcare, municipal social services, educational systems and disability interest organisations in the region where the study was conducted. Researchers 1 and 2 identified contact persons within identified municipal social services organisations, who identified eligible participants with ID. To facilitate the recruitment of individuals with ID, a short video was produced presenting the researchers, explaining the aim of the study, the purpose of the workshops and ethical considerations. The video, along with easy‐to‐read study information, was disseminated to contact persons with a request to present the material to potential participants with ID and to inquire about their interest in taking part in the study. Potential participants with ID expressed their interest in participating to the contact persons who notified Researcher 1 or 2.

Contact persons within identified healthcare, municipal social services, educational systems and disability interest organisations were identified and contacted directly by Researchers 1 and 2 through email, telephone calls or physical meetings. Study invitations and written information about the study were then distributed to managers within identified healthcare, municipal social services and educational systems organisations and contact persons within identified disability interest organisations. Managers and contact persons distributed the study invitation and written information to eligible participants within their organisations. Interested significant others and professionals contacted one or two who then emailed further information.

### 2.3 Participants

In total, 30 participants agreed to participate in the study: 14 individuals with ID, five significant others and 11 professionals. See Table 1 for participant characteristics.

**TABLE 1:** Participant characteristics.

Individuals with ID (*n* = 14)	
Mean age (min–max)	41.6 (16–67)
Gender	(*n*)
Female	11
Male	2
Non‐binary	1
**Significant others (*n* = 5)**	
Mean age (min–max)	52.2 (32–63)
Gender	(*n*)
Female	5
**Professionals (*n* = 11)**	
Mean age (min–max)	40.5 (26–58)
Gender	(*n*)
Female	11
Mean years of clinical experience (min–max)	13.2 (5–38)
Distribution of professions	(*n*)
Healthcare	
Occupational therapist	1
Physiotherapist	1
Social services	
Support worker	5
Educational systems	
High school teacher, higher education teacher and researcher	4

### 2.4 Data Collection

Workshops were employed, anticipated to support the generation of rich, contextually grounded insights in relation to the research question (Ørngreen and Levinsen 2017). Themes discussed were: needs, facilitators and barriers to health promotion; physical, mental and social well‐being; participation and relationships; health promotion through eHealth; and core components of digital intervention. The structure, format and content are further described in a protocol article (Müssener et al. 2023).

Eight workshops with three to nine participants were conducted between 2 May and 20 June 2023. Individuals with ID participated in two constellations: One group attended a series of three workshops, whereas the other group attended a single workshop. Significant others and professionals within healthcare, social services and educational systems were divided into four separate groups, attending one workshop each. Workshops were conducted in meeting rooms convenient for the participants, which were at Linköping University (*n* = 4), an after‐school club (*n* = 3) and an upper secondary school (*n* = 1).

Workshops lasted from 1 h 22 min to 3 h 40 min, including breaks. At the beginning of each workshop, participants and researchers introduced themselves, participants were informed about the study's aim and the proposed agenda, structure and topics for discussion. Researchers 2 and 3 actively participated in the workshops, whereas Researcher 1 was observing, taking field notes and asking supplementary questions. Field notes were taken both during and immediately after all workshops to document contextual details, observations and initial reflections. All workshops were audio‐recorded and professionally transcribed verbatim, with all identifying details anonymised for confidentiality.

### 2.5 Data Analysis

Data were analysed using qualitative content analysis, following an inductive approach to gain an understanding of strategies and organisational prerequisites for health promotion targeting individuals with ID as perceived by key stakeholders, including individuals with ID. In line with the approach described by Graneheim, Lindgren and Lundman (Lindgren et al. 2020; Graneheim et al. 2017), qualitative content analysis was conducted as a nonlinear process involving two overlapping and iterative phases: de‐contextualisation and re‐contextualisation. Both manifest and latent content were considered, and the level of abstraction and interpretation varied throughout the analytic process.

Initially, Researchers 4 and 1 thoroughly read all transcripts, verifying them against the audio recordings and making minor corrections. Researcher 4 then reread the transcripts multiple times to become familiar with and immersed in the data. In the phase of de‐contextualisation, meaning units relevant to the study aim were identified and highlighted and subsequently transferred to Microsoft Excel. Researcher 4 initially selected the meaning units, which were then discussed and confirmed with Researcher 1. The meaning units were then condensed while preserving core content and labelled with descriptive codes, closely aligned with the original text, using a low level of abstraction. Researchers 4 and 1 undertook condensation and coding, discussing it with all authors. Notes and initial reflections were documented throughout the process.

In the phase of re‐contextualisation, the codes were compared and sorted based on similarities and differences, then abstracted into broader categories. This step involved varying levels of abstraction, with a generally low degree of interpretation. Researcher 4 led the categorisation process in close collaboration with Researcher 1, and the analysis was regularly reviewed and discussed by the full research team. The process resulted in three categories, each described with a heading reflecting the manifest content, grounded in a transparent and collaborative analytic approach. See Table 2 for illustrative examples from the analysis process.

**TABLE 2:** Examples from the analysis process.

Meaning unit	And then the ice cream truck came on a Friday. We [staff at a group/service home] have a colleague who said, no, you bought ice cream last Wednesday. You do not need to buy any ice cream today. Get the credit card out! he said. So, there was ice cream anyway. He has realised that he can actually eat ice cream, and we cannot say no. We can maybe steer a little bit in one direction what they… You do not need to buy three kilos of ice cream.	I [individual with ID] want to be asked by the staff what I want… I want staff who are interested in me as a person.	They [staff at group/service home] had absolutely no knowledge regarding the diagnosis. It was stated in the service agreement, but they did not deliver at all.
Condensation	The ice cream truck came, and a colleague said no to buy ice cream. He got his credit card and bought ice cream anyway. He realised he can eat ice cream, and we do not have a say in it. We can steer a little bit, you do not need to buy three kilos of ice cream.	Want to be asked what I want, and to have staff who are interested in me.	Staff at group/service home had no knowledge regarding diagnosis, even though it was stated in the service agreement.
Code	Decide for yourself ice cream	Interested staff	Service agreement knowledge
Category	Enabling informed decision‐making for health promotion	Promoting health and well‐being through encouraging relations	Organisational factors influencing prerequisites for health promotion

## 3 Results

The result of the analysis is presented as three categories: enabling informed decision‐making; promoting health and well‐being through encouraging relations; and organisational structures influencing prerequisites for health promotion. These categories are derived through the lived experiences of individuals with ID, their significant others and professionals, offering valuable insights into how health promotion can be understood and supported in practice. Representative quotations from the transcribed text are presented in Boxes 1, 2, 3 to illustrate the results. ‘//’ denotes that the quotation has been shortened for readability. The designation after each quotation represents the participant's identification number, followed by the label ‘individual with ID’, ‘significant other’ or ‘healthcare, social services, or educational systems professional’.

**Box 1:** Representative quotations from the transcribed text.

Category: Enabling informed decision‐making for health promotion
Individual with ID: *The staff [at group/service home] cook breakfast, lunch, and dinner*. Moderator*: How interesting. Do you get to be involved in deciding what to cook?* Individual with ID: *Sometimes. We eat dinner at five o'clock*. Moderator: *Do you get to be involved in deciding what meals to cook, or do the staff decide?* Individual with ID*: The staff*. Moderator: *What do you think about that? Is it okay?* Individual with ID: *Yes*. Moderator: *Or does it feel boring?* Individual with ID: *No, I do not think so*. Participant 11 ‘individual with ID’ *What I primarily want to help them [individuals with ID] with is to understand their decision. Of course, they can buy ice cream and eat whatever they want. That is very important, and we need to somehow create conditions for them [to understand their decision], but that part about helping them understand [their decision], I think, is a huge challenge*. Participant 25 ‘social services professional’ Moderator: *How did you learn, about what is healthy to eat and so on?* Individual with ID: *Yes, I have learned it*. Moderator: *Have you received help from someone?* Individual with ID: *I have received help from someone*. Moderator: *From some staff [at group/service home] or some nurse?* Individual with ID: *Both a nurse and staff*. Participant 13 ‘individual with ID’ *I think it [step‐counting competition over three weeks] was good for some.//Some really went for it, or at least one individual [with ID] did, really all in. And he did not stop. So, it is probably important to think that if we do it another year, maybe we should limit it.//If you walk like he did, over 30 000 steps in a day, you walk quite a lot. And if you keep it up for a long time, it becomes a habit and then it can be hard to break that habit*. Participant 29 ‘educational systems professional’

**Box 2:** Representative quotations from the transcribed text.

Category: Promoting health and well‐being through encouraging relations
Moderator: *If you are going to use communication aids to promote an activity, is there any specific approach required?* Healthcare professional: *I think it is about showing that I am curious about what you [individual with ID] want and experience, and really delivering that feeling. You are not here to get information from me about what I am going to talk about. Instead, I want to know your challenges or interests, or whatever it is we are going to talk about today, and keep it open for anything that might come up. That is how it is when working with images [visual support]. It can feel a bit limiting because you [professional] need to prepare and bring everything with you. But also work with blank images and be able to fill in what comes up, so that you really can help to map out the whole situation of what we [individual with ID and professional] are talking about*. Participant 24 ‘healthcare professional’ Significant other: *We have asked [individual with ID] many times in various contexts. We always get the same answer, kind and nice. It may sound like quite soft, cautious, almost a bit meek qualities to have. But it is not, there are a lot of substance in it*. Moderator: *If you are kind and nice, you can refine*. Significant other: *Then you have a good approach*. Participant 16 ‘significant other’ *If you [individual with ID] have a bad experience from compulsory school, you carry it with you. You think it will be the same. We [professionals at upper secondary school] work a lot with the idea that this is a new chapter. Here, you have the opportunity to start from scratch and find your challenges. It is very much about slowly but surely finding each individual's own little challenge. Imagining it as a staircase, and leading the individual up this staircase, and then suddenly, wow, you did this. To see it and be like, oh! It is about having just the right kind of challenges and demands on oneself, and knowing what the expectations are*. Participant 20 ‘educational systems professional’ *My son [with ID] finds it very important to be involved and understand what is expected of him as a participant. He loves participating in activities and sports and such//but also preparation for participation. It is very important for individuals [with ID] to understand when and how things will happen. Not just being told or having it written down but really having the opportunity to understand. This also means, from my perspective, a lot of repetition. This applies to health and everything it entails. For him to understand, it requires, as in all situations really, a lot of repetition and consistency in trying to help him understand*. Participant 17 ‘significant other’ *We have been part of technological development. What is needed, again, unfortunately for this research project, is humans. It is the person‐to‐person contact that makes life dignified for individuals with ID, with the help of technology*. Participant 15 ‘significant other’

**Box 3:** Representative quotations from the transcribed text.

Category: Organisational factors influencing prerequisites for health promotion
Moderator: *Can you eat in the common area where you live [at a group/service home]?* Individual with ID: *Yes, if I wanted to. But I cannot, because they [staff at group/service home] must give me the food*. Moderator: *What did you say now? What have they done?* Individual with ID: *[External provider] does not want them [staff] to cook*. Moderator: *So, [external provider] is the one who manages your [group/service] home? And they [staff] are not allowed to cook?* Individual with ID: *After all these years*. Moderator: *But they [staff] did it before, right?* Individual with ID: *Yes, before [external provider] took over*. Moderator: *I find that interesting. So, they [staff] do not cook in the common area anymore?* Individual with ID: *No, not now when they [external provider] have taken over*. Moderator: *So, they [external provider] deliver ready‐to‐eat meals, or?* Individual with ID: *Yes*. Moderator: *What do you think about that?* Individual with ID: *That is why I would like to cook healthier meals*. Participant 10 ‘individual with ID’ *I am working [at a daily activity centre] with an individual [with ID] who has a huge problem with the intake of unhealthy food. We are talking about three, four litres of Coca‐Cola every day. It is crisps. It is pan pizzas. It is such extreme amounts. It is not even a little abnormal, it is extreme. I talked to the staff at the group/service home and said, you have to do visual support. I work a lot with social stories. I said, make a social story, explain what it [the problem] is. They said, no, we are not allowed to do that. I almost get the feeling that some staff [at group/service homes] think it is quite nice that this law [the LSS Act] on self‐determination exists, so they can disclaim some of the responsibility*. Participant 22 ‘social services professional’ *I also noted that the staff [at group/service homes] must//have knowledge about health and what we generally consider health. What the individual [with ID] sees as health. [Individual with ID] would never be able to manage it himself and say, I need this because… He does not have that ability, he learns over time. If staff said he could choose freely, he would think it was really cool to eat candy every day or use the tablet all day. So, with structure, you need staff who know that even if it is a bit boring now, we are doing it anyway, and motivates. But those [staff] who are going to motivate must understand why things are done. And it is, again, about education, information and knowledge. If staff is to guide an individual [with ID] who does not quite have the ability themselves, if that staff does not understand and have knowledge, how will these individuals [with ID] be given the opportunity? I do not see that as very clear. They [individuals with ID] must be guided by those who can*. Participant 17 ‘significant other’

### 3.1 Enabling Informed Decision‐Making for Health Promotion

This category illustrates how enabling informed decision‐making, by supporting individuals with ID to gain knowledge about healthy living, their health situation and behaviours and consequences of unhealthy decisions, is central to health promotion. At the same time, findings show that support persons struggle with balancing how much support to give without compromising the autonomy of individuals with ID regarding healthy decisions.

All participant groups reflected on the significance of choice for individuals with ID, discussing varying degrees of involvement in decision‐making. Some participants with ID were content with staff at group and service homes making decisions on their behalf, whereas others expressed a desire for more involvement in decisions. For example, some valued being able to choose what to cook and eat themselves, whereas others were comfortable with staff working at group and service homes to make those decisions for them. Professionals within healthcare, social services and educational systems reflected on the balance between supporting individuals with ID in making healthy choices while respecting their preferences and ability to make their own decisions. They emphasised the importance of enabling individuals with ID to make informed and healthy decisions based on health‐related knowledge rather than imposing restrictions or prohibiting specific lifestyle behaviours.

This was expressed as a need to support individuals with ID in learning what constitutes a healthy lifestyle and understanding the potential health consequences of unhealthy choices. Participants with ID shared examples of being supported by staff at group and service homes and healthcare professionals in learning about healthy eating. However, both participants with ID and professionals acknowledged that in certain contexts, limiting choices could support health promotion. For instance, providing a manageable number of options was seen as essential, as too many choices could lead to anxiety, overeating or sedentary behaviour.

### 3.2 Promoting Health and Well‐Being Through Encouraging Relations

This category demonstrates how encouraging relations serve as a supportive strategy in health promotion. Significant others and professionals stressed that it is important to provide individuals with ID with the ability to express themselves and to be heard and understood. Professionals underscored the importance of adapting communication to the needs of the unique individual with ID, using simplified language and communication aids such as talking mats, social stories, visual support, sign‐supported speech and motivational interviewing. These communication aids help individuals with ID to better express their interests and needs, and professionals shared examples of enabling individuals with ID to express their interests and incorporate these into communication aids. They emphasised that demonstrating genuine interest in individuals with ID is a way to foster trust and engagement.

Personal attributes and demeanour emerged as another key theme. All participant groups emphasised the importance of support persons being humorous, cheerful, positive, kind and pleasant in interactions with individuals with ID. Although such qualities were seen as supportive, professionals within educational systems also pointed out the necessity of maintaining boundaries, that is, the need to balance these personal attributes with clear professional boundaries.

All participant groups also underscored the importance of experiences that build confidence, such as overcoming challenges and achieving success. These experiences were seen as vital for individuals with ID to be empowered in taking on new health‐related challenges. At the same time, professionals acknowledged that demands and expectations could contribute to health promotion, provided that they were tailored to the individual with ID's abilities and supported by clear communication. For instance, all participant groups described how preparing individuals with ID for upcoming events and clarifying expectations were essential for fostering success.

Technological devices for encouraging physical activity were discussed by all participant groups, including game applications, step counters and fitness trackers. Although these devices were seen as motivating, especially among individuals with ID, significant others and professionals stressed that technology alone was insufficient for encouraging physical activity. Personal support was deemed critical to ensure sustained engagement and progress. Additionally, social services professionals shared examples of integrating physical activity into routines and work assignments at daily activity centres as useful for encouraging health in a natural way. Finally, professionals highlighted the value of continuous support and tangible rewards in health promotion. Examples included providing consistent guidance over time and celebrating achievements with diplomas or medals. These strategies were seen as reinforcing positive behaviours and promoting a sense of accomplishment.

### 3.3 Organisational Structures Influencing Prerequisites for Health Promotion

This category points to the role of organisational structures in shaping opportunities and constraints for support persons to provide support to individuals with ID in health‐related settings. Significant others and social services professionals described how organisational structures influence health promotion efforts targeting individuals with ID in everyday practice. According to significant others, one factor was that service agreements prioritised cost‐effectiveness and logistical convenience, sometimes at the expense of the quality of support provided to individuals with ID. For example, rather than preparing meals on‐site, some group and service homes rely on external providers to deliver ready‐to‐eat meals, which were perceived by significant others as more cost‐efficient due to staff shortages. This approach not only limits the opportunity for individuals with ID to engage in cooking and meal preparation but also restricts the level of personal support available to them.

Furthermore, a factor acting as a significant barrier was the lack of clear routines and clearly defined responsibilities within group and service homes, particularly evident in the dissemination of information related to health and leisure activities. According to significant others, without well‐established routines or clear communication channels, individuals with ID often miss out on health‐promoting activities, limiting their opportunities for engagement and participation in well‐being‐enhancing experiences.

Additionally, another challenge that emerged relating to the organisation of group and service homes was a tension between health promotion efforts and legislation, as this legalisation emphasises the individual with ID's right to self‐determination and integrity, as well as their right to influence the services provided. Significant others and professionals representing healthcare and social services shared experiences of situations where they felt that staff at group and service homes invoked the right to self‐determination to justify not supporting individuals with ID, despite evident health problems and unhealthy lifestyle behaviours.

Significant others and professionals attributed the lack of support for individuals with ID to a lack of knowledge on healthy behaviours among staff within the organisations. This included negative attitudes towards healthy diets and physical activity, as well as a lack of knowledge about how to support individuals with ID in health promotion. This lack of support results in individuals with ID missing out on health promotion efforts, negatively affecting their health.

## 4 Discussion

The findings from this qualitative study indicate that identified strategies and organisational structures are intertwined in everyday health promotion targeting individuals with ID. This study shows that the strategy enabling informed decision‐making based on health‐related knowledge is central to promoting health among individuals with ID. Although no explicit questions about learning were asked during the workshops, professionals expressed a need to support individuals with ID in learning what constitutes a healthy lifestyle and understanding the potential health consequences of unhealthy lifestyle choices and participants with ID shared examples of being supported by staff at group and service homes and healthcare professionals in learning about health, for example, healthy eating.

These findings underscore that learning among individuals with ID is an integral component of health promotion for this population, mirroring its importance in health education for the general population (World Health Organization [WHO] 2021). Previous research similarly demonstrates this, for example, through a multicomponent intervention in which recurring health education sessions contributed to improved physical activity among individuals with ID (Bergström et al. 2013).

Findings from this study demonstrate that encouraging relations serves as a supportive strategy for promoting health and well‐being among individuals with ID. Conclusions from this study must be interpreted in light of the fact that no explicit questions about learning were asked during the workshops. Nevertheless, it is still interesting to consider whether identified strategies may also support learning among individuals with ID in health promotion. The findings show that significant others and professionals stressed the importance of enabling individuals with ID to express themselves and to be heard and understood. Professionals underscored the importance of adapting communication to the needs of the unique individual with ID, using simplified language and communication aids. This strategy aligns with teaching and pedagogical strategies described in previous research such as using talking mats (Martin et al. 2021), pictures and other communicative support (Bergström et al. 2014) to facilitate learning among individuals with ID.

Findings also reveal that all participant groups emphasised the importance of supporting individuals with ID in overcoming challenges and achieving success in health promotion. For example, participants described doing preparatory efforts for upcoming events and clarifying expectations as essential for fostering success. Moreover, professionals highlighted the value of providing consistent guidance over time and celebrating achievements with diplomas or medals in health promotion targeting individuals with ID. These strategies align with previous research describing pedagogical strategies, such as doing preparatory efforts, encouraging participation in the health course activities, offering assistance when needed and using rewards (Bergström et al. 2014).

This study also disclosed that organisational structures influenced everyday health promotion efforts targeting individuals with ID. For example, significant others and healthcare and social services professionals shared experiences of situations where they felt that staff at group and service homes invoked the right to self‐determination to justify not supporting individuals with ID, despite evident health problems and unhealthy lifestyle behaviours. Although they attributed this to a lack of knowledge on healthy behaviours among staff within the organisations in this study, previous research has shown that support persons face dilemmas when it is unclear whether unhealthy decisions are the responsibility of individuals with ID or their support persons (Overwijk et al. 2021).

This dilemma highlights the necessity for enhanced education, knowledge and training regarding healthy behaviours and how to support individuals with ID in health promotion and informed decision‐making within organisations that serve this population. This is in line with previous research identifying a lack of formal education regarding both ID and general health promotion and disease prevention among healthcare and social care professionals (Vi et al. 2023; Patja et al. 2022). This educational gap contributes to unmet healthcare needs and health inequalities for individuals with ID (Björne and Flygare Wallén 2024). This is concerning as it is well known that individuals with ID need support to facilitate a healthy lifestyle (Leser et al. 2018; Kuijken et al. 2016; Nijhof et al. 2024; Hewitt et al. 2013; Roll 2018; Overwijk et al. 2021; Kuijken et al. 2019).

Based on the findings in this study, there appears to be a critical need for enhanced education and knowledge for healthcare and social services professionals to encounter and support individuals with ID in health promotion (Leser et al. 2018; Kuijken et al. 2016; Nijhof et al. 2024; O'Leary, Taggart, and Cousins 2018; Kuijken et al. 2019). This education could be integrated at both the pre‐graduate and graduate levels, as well as through in‐service training and continuing professional development. Further research is needed to investigate the development and integration of such educational initiatives within organisations that provide health‐promoting support to individuals with ID, ensuring these effectively contribute to continuous professional development in healthcare and social services.

By including the perspectives of individuals with ID, their significant others and professionals, this study recognises a need for learning and understanding among individuals with ID to make informed healthy decisions and indicates that their support persons play a facilitating role in this process. The study also demonstrates the importance of support persons fostering encouraging relations to promote health and well‐being among individuals with ID. Although examples show support persons successfully promoting health, the findings also point to the need for enhanced education and knowledge among professionals providing health‐promoting support in everyday contexts.

### 4.1 Strengths and Limitations

Several methodological strategies were taken to ensure trustworthiness (Stenfors et al. 2020; Korstjens and Moser 2018). The qualitative study design was well‐suited for gaining insights into and valuing the participants' own experiences and perspectives. However, the small number of male participants and their uneven distribution across groups constitute a limitation. This imbalance reduced the diversity of gender perspectives, meaning that the male perspective is not fully represented in this study, which has implications for the interpretation of findings and affects the credibility (Stenfors et al. 2020; Korstjens and Moser 2018). This imbalance may result in the study sample differing from the broader population, affecting the transferability (Stenfors et al. 2020; Korstjens and Moser 2018).

Although the study has limitations, credibility (Stenfors et al. 2020; Korstjens and Moser 2018) was strengthened through the inclusion of both individuals with ID and support persons representing different ages, experiences, expertise and perspectives. To enable readers to assess transferability (Stenfors et al. 2020; Korstjens and Moser 2018), participant characteristics are described in the Methods section. The transferability may vary depending on the context, depending on how the healthcare and social services are financed and organised. Therefore, a thorough description of the study context is provided in the Methods section. Despite these limitations, the results are considered relevant and applicable to similar settings.

The sample size was based on information power (Malterud et al. 2016), and the data collection ended when the data reached satisfactory depth, richness, and appropriateness to answer the research question with sufficient confidence. A limitation is that data on recruitment attrition were not collected from those assisting with recruitment in order to avoid placing additional demands on their working time. As a result, it is not possible to determine how many individuals declined participation or the reasons for non‐participation.

To achieve dependability (Stenfors et al. 2020; Korstjens and Moser 2018), the research process was thoroughly documented following the COREQ checklist (Tong et al. 2007), creating a clear audit trail. Data collection and analysis were grounded in literature (Lindgren et al. 2020; Graneheim et al. 2017; Müssener et al. 2023) to ensure a rigorous and systematic process. Researcher triangulation during analysis added credibility and reduced the risk of one researcher's subjective interpretation dominating the analysis (Patton 2015). Reflexivity (Stenfors et al. 2020; Korstjens and Moser 2018) was addressed through ongoing discussions about how researchers' previous knowledge and experiences might have influenced both data collection and interpretation. The results were discussed in relation to previous research and theory, also enhancing the credibility (Patton 2015; Stenfors et al. 2020). Quotations from transcriptions were included to demonstrate transparency and enhance confirmability (Stenfors et al. 2020; Korstjens and Moser 2018), with unique identifiers for each participant to demonstrate that various participants were represented across the results.

## 5 Conclusions

This study highlights the relevance of recognising learning among individuals with ID as a vital component of everyday health promotion. Findings show that the strategy of enabling informed decision‐making relies on supporting individuals with ID in learning and understanding health and healthy behaviours, with support persons playing a central role in facilitating health‐related learning. Supportive approaches embedded in the strategy encouraging relations enable communication, motivation, and engagement in everyday contexts. Importantly, these strategies are inseparable from organisational structures, which affect the prerequisites for everyday health promotion targeting individuals with ID.

## Funding

This research was funded by the Swedish Research Council (Vetenskapsrådet) under grant number 2022‐06287, the Swedish Research Council for Health, Working Life and Welfare (Forte), under grant number 2024‐02286 and the Sunnerdahl Handikappfond Foundation (Stiftelsen Sunnerdahls Handikappfond) under grant number F27/22.

## Ethics Statement

This study received ethical approval from the Swedish Ethical Review Authority under reference number 2022‐06682‐01. All participants provided written informed consent.

## Conflicts of Interest

The authors declare no conflicts of interest.

## Data Availability

The dataset is available from the corresponding author, but restrictions apply to the availability of the data to protect the study participants' privacy. Pseudo‐anonymised data are available upon reasonable request.
